# Gold-catalyzed naphthalene functionalization

**DOI:** 10.3762/bjoc.7.77

**Published:** 2011-05-23

**Authors:** Pedro J Pérez, M Mar Díaz-Requejo, Iván Rivilla

**Affiliations:** 1 Laboratorio de Catálisis Homogénea, Departamento de Química y Ciencia de los Materiales, Unidad Asociada al CSIC, Centro de Investigación en Química Sostenible (CIQSO), Universidad de Huelva, Campus de El Carmen 21007-Huelva, Spain

**Keywords:** carbene insertion, copper catalysts, diazoacetates, gold catalysts, naphthalene functionalization, selective insertion

## Abstract

The complexes IPrMCl (IPr = 1,3-bis(diisopropylphenyl)imidazol-2-ylidene, M = Cu, **1a**; M = Au, **1b**), in the presence of one equiv of NaBAr'_4_ (Ar' = 3,5-bis(trifluoromethyl)phenyl), catalyze the transfer of carbene groups: C(R)CO_2_Et (R = H, Me) from N_2_C(R)CO_2_Et to afford products that depend on the nature of the metal center. The copper-based catalyst yields exclusively a cycloheptatriene derivative from the Buchner reaction, whereas the gold analog affords a mixture of products derived either from the formal insertion of the carbene unit into the aromatic C–H bond or from its addition to a double bond. In addition, no byproducts derived from carbene coupling were observed.

## Introduction

**Scheme 1:**
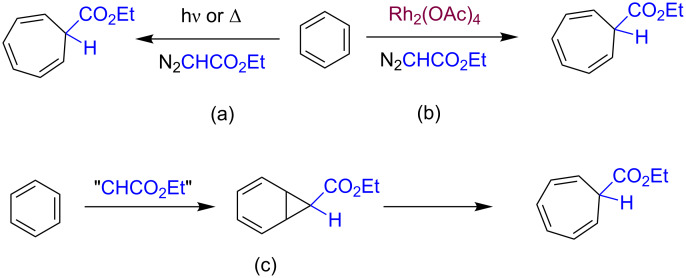
(a) The Buchner reaction of benzene and ethyl diazoacetate and (b) the Rh-catalyzed version. (c) Both pathways involve the formation of norcaradiene intermediates.

At the end of the nineteenth century, Buchner discovered [1] the thermal and photochemical route for the functionalization of benzene using diazo compounds to provide a carbene moiety. The first step of this transformation consists of the addition of such a unit to the aromatic double bond to give a norcaradiene intermediate that spontaneously undergoes ring opening to afford the more stable cycloheptatriene product (Scheme 1) [2]. Nearly one century later, Teyssié and co-workers discovered the potential of dirhodium tetraacetate and related Rh_2_(L-L)_4_ compounds as catalysts for the decomposition of diazo compounds and subsequent transfer of the carbene moiety to several saturated and unsaturated substrates, including aromatics [3]. Thus, the reaction of ethyl diazoacetate (EDA) with benzene in the presence of such catalysts at room temperature exclusively affords the cycloheptatriene product in quantitative yields. The reaction, always referred to the intermolecular version, was later observed with other metal-based catalysts [4–6].

**Scheme 2:**
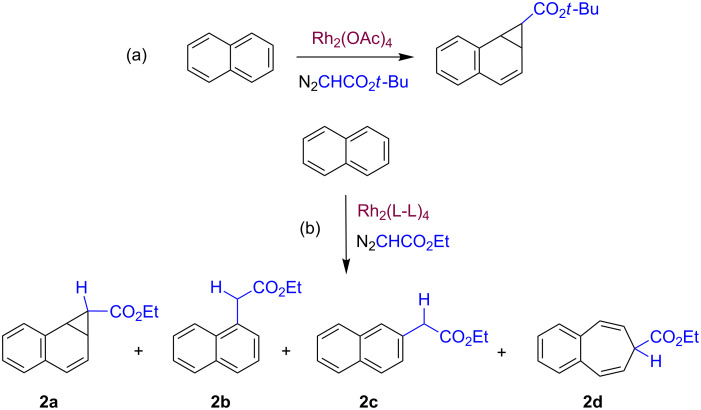
The Buchner reaction applied to naphthalene. (a) Teyssié's system. (b) Müller's system.

The above transformation with rhodium-based catalysts [7–8] has also been investigated with naphthalene as a substrate. In this case, Teyssié and co-workers showed that it could be converted, using *t*-butyl diazoacetate, into norcaradiene type derivatives, formed by the cyclopropanation of one of the double bonds of the naphthalene ring. Later, Müller and co-workers [9] showed the effect of a series of Rh_2_(L-L)_4_ in the same transformation but with ethyl diazoacetate as the carbene source. A mixture of the products (**2a**–**d**) arising from cyclopropanation, ring opening and the formal insertion of CHCO_2_Et into the aromatic C–H bonds were observed, with **2a** being by far the major product (Scheme 2).

**Scheme 3:**
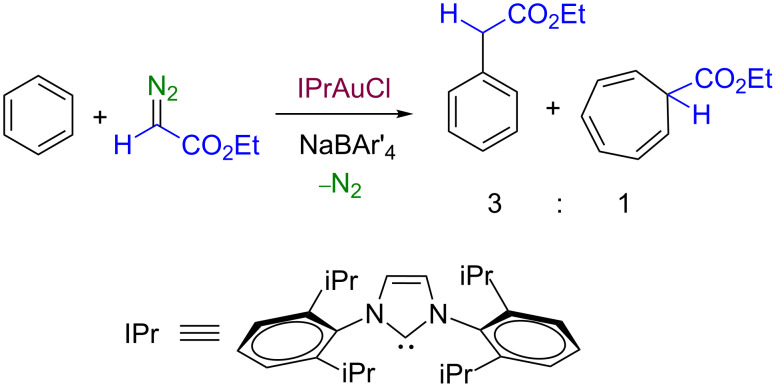
The gold-catalyzed reaction of benzene and EDA.

In the course of our research, focussed on the development of group 11 metal-based catalysts for carbene transfer reactions from diazo compounds [10], we found that the gold complex IPrAuCl (**1b**) (IPr = 1,3-bis(diisopropylphenyl)imidazol-2-ylidene) in the presence of one equiv of NaBAr'_4_ (Ar' = 3,5-bis(trifluoromethyl)phenyl) induced the functionalization of benzene with ethyl diazoacetate to give a mixture of a cycloheptatriene and ethyl 2-phenylacetate [11], the latter being the result of the formal insertion of the CHCO_2_Et group into the C–H bond of benzene as well as the major product (Scheme 3). In this contribution, we report the results obtained from the analogous transformation using naphthalene as the substrate, with copper- and gold-based catalysts, not previously described for the functionalization of such fused arenes.

## Results and Discussion

### Reaction of naphthalene and diazoacetates catalyzed by IPrMCl (M = Cu, Au)

**Scheme 4:**
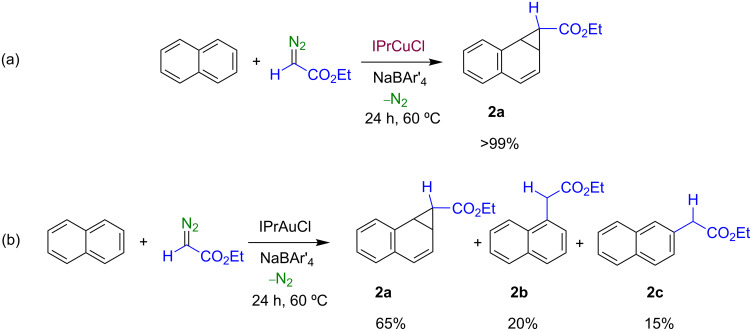
The functionalization of naphthalene with ethyl diazoacetate catalyzed by the complexes (a) **1a** and (b) **1b**.

When dichloroethane solutions of naphthalene were treated with ethyl diazoacetate in the presence of catalytic amounts (5%) of a 1:1 mixture of IPrMCl (M = Cu, **1a**; M = Au, **1b**) and NaBAr'_4_, the diazo compound was consumed after 24 h at 60 °C (no significant reaction was observed at room temperature or at 40 °C). NMR analysis of the crude reaction product revealed that when **1a** was used as the catalyst, only one compound was formed, identified as ethyl 1a,7b-dihydro-1H-cyclopropa[a]naphthalene-1-carboxylate (**2a**), i.e., the product derived from the direct cyclopropanation of the naphthalene C–C double bond (Scheme 4a). By contrast, the use of the gold catalyst IPrAuCl (**1b**) under the same reaction conditions gave a mixture of three compounds, in ca. 65:20:15 ratio, that have been identified as **2a**, ethyl 2-(naphthalen-1-yl)acetate (**2b**) and ethyl 2-(naphthalen-2-yl)acetate (**2c**). Compounds **2b** and **2c**, respectively, are derived from the formal insertion of the carbene group into a C–H bond of naphthalene (Scheme 4b). The selectivity observed is similar to that reported by Müller's group with [Rh_2_(O_2_CC_3_F_7_)_4_] (60:22:18) [8]. However, in our case the yield of products (EDA-based) was quantitative: products derived from dimerization of the diazo compound, i.e., diethyl fumarate and maleate, were not detected. The absence of the fused cycloheptatriene **2d** in our system is also noteworthy. Substituted naphthalenes with OMe or Cl substituents at the beta position were also employed as substrates, however, the yields of the desired products were nearly negligible. The former seemed to induce the insertion into the Me groups, whereas in the latter case the aromatic reagent seemed deactivated.

**Scheme 5:**
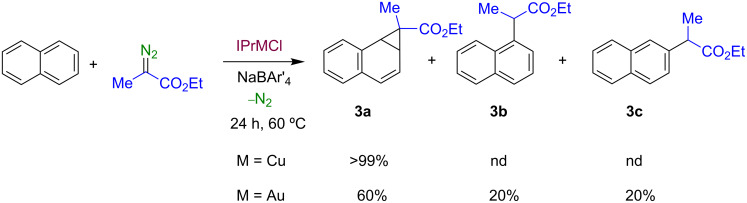
The functionalization of naphthalene with ethyl 2-diazopropionate catalyzed by complexes **1a** and **1b**.

We have expanded this reaction to ethyl 2-diazopropionate as the diazo component. Following a similar protocol, naphthalene was reacted in dichloroethane with ethyl 2-diazopropionate in the presence of a 1:1 mixture of **1a**,**b** and NaBAr'_4_ (5% with respect to the diazo compound). Similarly to the previous results, the fused norcaradiene **3a** (Scheme 5) was exclusively and quantitatively formed using the copper catalyst **1a**, whilst the use of **1b** afforded a mixture of three products in a 60:20:20 ratio. The major product was identified as the **3a** and the minor products have been characterized as the insertion products of the carbene C(Me)CO_2_Et into the α- and β-C–H bonds of naphthalene, **3b** and **3c**, respectively. When other diazo reagents such as Me_3_SiC(N_2_)CO_2_Et or PhC(N_2_)CO_2_Et were employed, intractable mixtures of compounds, probably due to multiple insertions, were observed by NMR.

It is also worth mentioning that the above transformations do not compete with the formation of byproducts derived from the catalytic dimerization of the diazo reagents, a common drawback in this methodology [2]. Despite of adding all the diazo compound in one portion at the beginning of the reaction, the final reaction mixture only showed resonances due to the aforementioned insertion and addition products. This is at variance with other reported systems that required the use of slow addition devices to diminish the formation of such byproducts.

## Conclusion

The complexes IPrMCl (M = Cu, Au) catalyze the transfer of carbene groups C(R)CO_2_Et (R = H, Me) to naphthalene, in the presence of NaBAr'_4_ as halide scavenger, to give mixtures of products via carbene insertion into a C–H bond or by addition to a double bond. In the case of copper, norcaradiene type compounds are formed quantitatively. The use of the gold analogue also induces the formation of such fused cyclopropanes in addition to the products derived from the formal insertion of the carbene units into the C–H bonds of naphthalene. The system is completely chemoselective with regards to arene functionalization (with no diazo compound dimerization being observed).

## Experimental

All reactions and manipulations were carried out under a nitrogen atmosphere. Organic solvents were dried, distilled, and degassed before use. The reagents were purchased from Sigma Aldrich. Complexes IPrMCl (M = Cu, **1a**; M = Au, **1b**), NaBAr'_4_ and ethyl 2-diazopropionate were prepared by literature procedures [12–16]. ^1^H and ^13^C NMR spectra were recorded on a Varian Mercury 400 spectrometer in CDCl_3_ as solvent, with chemical shifts (δ) referenced to internal standards.

### General catalytic experiment

Complex **1** (0.025 mmol) was dissolved in dichloroethane (5 mL) and one equiv of NaBAr'_4_ added to the solution, which was then added to a solution of naphthalene (8.6 mmol, 10 mL) and heated at 60 °C in dichloroethane (20 mL). After stirring for 15 min, (R)C(N_2_)CO_2_Et (R = H, Me; 0.5 mmol) was added in one portion, and the mixture stirred for 24 h. Removal of volatiles followed by silica gel column chromatography (1:1 Et_2_O:petroleum ether) gave a mixture of products. The products **2a**, **3b** and **3c** were identified by comparison with literature data [17–19], and **2b** and **2c** were compared authentic samples obtained from commercial sources.

Spectroscopic data for **3a**: ^1^H NMR (400 MHz, CDCl_3_) δ 7.41–6.94 (m, 4H), 6.59 (d, 1H), 6.06 (dd, 1H), 4.23 (m, 2H), 3.14 (d, *J* = 8.7 Hz, 1H), 2.72 (dd, *J* = 8.8 Hz, 1H), 1.26 (s, 3H), 1.29 (m, 3H); ^13^C NMR (101 MHz, CDCl_3_) δ 169.0 (CO_2_Et), 146.4, 134.5, 133.4, 129.6, 128.5, 127.7, 126.6, 125.5 (aromatic), 63.1 (CO*C*H_2_CH_3_), 39.6, 33.3, 31.2 (cyclopropyl), 19.52 (CH_3_), 11.8 (COCH_2_*C*H_3_); MS *m*/*z* (%): 228 (70), 199 (30), 182 (100).
